# Active Poly(3-hydroxybutyrate-co-3-hydroxyvalerate) (PHBV) Films Containing Phenolic Compounds with Different Molecular Structures

**DOI:** 10.3390/polym16111574

**Published:** 2024-06-02

**Authors:** Carla Ivonne La Fuente Arias, Chelo González-Martínez, Amparo Chiralt

**Affiliations:** Institute of Food Engineering, FoodUPV, Universtitat Politècnica de València (UPV), 46022 Valencia, Spain; cgonza@tal.upv.es (C.G.-M.); dchiralt@upv.es (A.C.)

**Keywords:** PHVB, phenolic compounds, biodegradable packaging, catechin, ferulic acid, vanillin, overall and specific migration

## Abstract

To obtain more sustainable and active food packaging materials, PHBV films containing 5% wt. of phenolic compounds with different molecular structures (ferulic acid, vanillin, and catechin) and proved antioxidant and antimicrobial properties were obtained by melt blending and compression molding. These were characterized by their structural, mechanical, barrier, and optical properties, as well as the polymer crystallization, thermal stability, and component migration in different food simulants. Phenolic compounds were homogenously integrated within the polymer matrix, affecting the film properties differently. Ferulic acid, and mainly catechin, had an anti-plasticizing effect (increasing the polymer glass transition temperature), decreasing the film extensibility and the resistance to breaking, with slight changes in the elastic modulus. In contrast, vanillin provoked a plasticizing effect, decreasing the elastic modulus without notable changes in the film extensibility while increasing the water vapor permeability. All phenolic compounds, mainly catechin, improved the oxygen barrier capacity of PHBV films and interfered with the polymer crystallization, reducing the melting point and crystallinity degree. The thermal stability of the material was little affected by the incorporation of phenols. The migration of passive components of the different PHBV films was lower than the overall migration limit in every simulant. Phenolic compounds were released to a different extent depending on their thermo-sensitivity, which affected their final content in the film, their bonding forces in the polymer matrix, and the simulant polarity. Their effective release in real foods will determine their active action for food preservation. Catechin was the best preserved, while ferulic acid was the most released.

## 1. Introduction

The development of more sustainable materials for food packaging is necessary to reduce the environmental impact of single-use packaging. At the same time, the active character of these materials is of great interest to the food industry as they allow one to extend the shelf life of products, enlarging their distribution period and reducing food losses along the distribution chain. Poly(hydroxyalkanoates) are a wide class of linear polyesters belonging to bio-based and biodegradable thermoplastic polymers [1]. They are synthesized by a large number of microorganisms, resulting in polymers with great structural diversity [2]. In recent years, special attention has been paid to PHBV (poly(3-hydroxybutyrate-co-3-hydroxyvalerate)), which has better physical properties (toughness, flexibility, and processability) compared to other PHA polyesters [2]. This polymer could be used to obtain sustainable food packaging materials since it can be composted and incorporated into the organic matter cycle.

The incorporation of natural compounds with antioxidant and/or antimicrobial properties, such as phenolic compounds, into the polymer matrix may contribute to an improvement in the performance of the material properties, while the incorporated compounds provide the materials with a better food preservation capacity [3]. There is a wide range of phenolic compounds used for different applications, such as those with simple chemical structures, such as flavonoids, hydroxybenzoic acids, hydroxycinnamic acids, and complex polyphenols such as tannins (proanthocyanidins, gallotannins, ellagitannins) [1]. Some of these compounds have been used as food additives to obtain active materials for food packaging applications [4,5]. Nevertheless, the incorporation of phenolic compounds into polymer matrices requires the study of their influence on the properties of the materials and the consequent impact on their adequacy for food packaging applications, as well as the capacity to maintain activity in the different stages of the material production and use [1]. These aspects are greatly affected by the compound molecular structure, its ability to establish interchain interactions within the polymer matrix, and its thermo-sensitivity and stability that greatly affect its preservation during the material processing and storage. Likewise, the compound molecular structure greatly affects its ability to be released in contact with food since it is related to the bonding forces established within the polymer matrix and the solubility into the food system, both defining the compound’s partition coefficient.

From the available phenolic compounds with potential to produce active polymeric materials, ferulic acid is a hydroxycinnamic acid (3-(4-hydroxy-3-methoxyphenyl)prop-2-enoic acid) that has low toxicity and many physiological functions (anti-inflammatory, antioxidant, antimicrobial activity, anticancer, and antidiabetic effects) and has been widely used in the pharmaceutical, food, and cosmetics industry [6]. It is a free radical scavenger that inhibits the catalysis of the enzyme’s free radical generation [7]. Likewise, relatively low concentrations of this compound inhibited the growth of numerous bacteria [8]. Biodegradable PHBV films with ferulic acid with antibacterial properties have been previously studied [9].

Another interesting phenol that can be used to obtain active materials is vanillin (4-hydroxy-3-methoxybenzaldehyde). It is a white to mildly yellow crystalline powder with an interesting fragrance used as a food additive with multiple biological properties [10]. Different studies have shown that vanillin possesses an antimicrobial effect against yeasts, molds, and bacteria, with relatively low values of its minimum inhibitory concentration [11]. This compound also exhibited antimicrobial properties when incorporated into PHBV films while reducing the thermal stability of the polymer and the tensile strength of the films [12]. 

Catechin is a favan-3-ol belonging to the polyphenol subgroup called flavonoids that can be used to produce active films. The antioxidant and antimicrobial activities of catechin and derivatives have been described in several studies [13,14]. It has also been used to develop active films for food packaging based on PVA–starch blends that exhibited antimicrobial and antioxidant capacity to preserve raw beef [15]. This compound greatly modified the PHB structure due to the formation of hydrogen bonds, enhancing the interchain forces [16]. 

PHBV materials are authorized for food contact applications by the European Plastics Regulation (EU) No 10/2011 due to their inertness in liquid food simulants. However, few studies have been published about the effect of the phenolic compounds on the material properties when incorporated into the polymer matrix, as well as the migration of film components into the foodstuff, which is important for ensuring food quality and safety.

This study aims to obtain new insights about the impact of some phenolic compounds with different molecular structures, such as catechin, ferulic acid, and vanillin, on the functional and structural properties of PHBV films, their thermal stability, and crystallization, as well as their migration behavior into different food simulants in terms of the release of the passive components and potentially active compounds that will determine the food preservation capacity of the packaging material. Likewise, the preservation of phenolics during the thermal processing of the films was analyzed. The information obtained will allow for a better choice of the active compound based on its different thermo-sensitivity during the thermoprocessing of the material and its release capacity depending on the binding forces on the polymeric matrix.

## 2. Materials and Methods

### 2.1. Materials

Poly(3-hydroxybutyrate-co-3-hydroxyvalerate) (Enmat Y1000P PHBV, melting point: 175–180 °C, Co-polymer content: 1–2%, heat deflection temperature (HDT)—ISO 7619 [17]: 157–165 °C, density 1.23 g/cm^3^) was supplied by Helian Polymers B.V. (Belfeld, Holland). (±)-Catechin (98% purity) was supplied by Biosynth (Bratislava, Slovakia) and ferulic acid (99% purity) and vanillin (99% purity) were supplied by Sigma Aldrich (Barcelona, Spain). Other reactants, phosphorus pentoxide (P_2_O_5_), methanol, and ethanol were supplied by Panreac Química, S.A. (Barcelona, Spain).

### 2.2. Film Preparation

PHBV films were prepared with active compounds such as catechin, ferulic acid and vanillin at a concentration of 5 g/100 g of blend with the polymer. The phenol concentration was chosen based on previous studies [9], with the minimum concentration possible in order not to alter the properties of the polymer too much but to ensure the necessary release for achieving an effective concentration on the packaged food surface. Firstly, PHBV was dried at 60 °C for 24 h to remove water to avoid hydrolysis during heating. The PHBV pellets were cold-powdered and homogenously mixed with the corresponding amount of the phenol powder before melt blending. Then, blends were melt-blended using an internal mixer (HAAKETM PolyLabTM QC, Thermo Fisher Scientific, Dreieich, Germany) at 170 °C and 50 rpm for 12 min. These blends were cold-milled (Thermomix Tm31, Vorwerk, Barcelona, Spain) to obtain powder. The films were obtained in a hot-plate hydraulic press (Model LP20, Labtech Engineering, Bangkok, Thailand) using 3.3 g of the blend powder per film. The sample was placed onto a PTFE sheet in the press, pre-heated at 180 °C for 5 min, compressed at 180 °C and 100 bars for 4 min, and, finally, cooled to 70 °C for 3 min. All films were conditioned at 0% relative humidity (RH) (in P_2_O_5_) or 53% RH (in over-saturated Mg (NO_3_)_2_) until further analyses.

### 2.3. Morphology

The film cryo-fractured cross-sections were analyzed using a field emission scanning electron microscope (ULTRATM 55, Zeiss, Oxford Instruments, Oxford, UK). The samples were covered with platinum, and the micrographs were obtained at an acceleration voltage of 2.00 kV.

### 2.4. Mechanical Properties

A universal testing machine (Stable Micro Systems, TA.XT plus, Hasmelere, UK) was used to carry out the tensile test in films conditioned at 53% RH, determining tensile strength at break (*TS*), elongation at break (*ε*), and the elastic modulus (*EM*), following ASTM D882-12 [18]. At least eight samples with dimensions of 25 × 10 mm^2^ were evaluated for each formulation. The initial grip separation was 50 mm, and the crosshead speed was 12.5 mm·min^−1^. The film thickness was determined at five random positions using a digital micrometer (±0.001 mm).

### 2.5. Water Vapor Permeability

The water vapor permeability (WVP) was determined in triplicate, in films, using the gravimetric method following ASTM E96/E96M [19] and the correction proposed by McHugh [20]. The film samples (Ø = 3.5 cm), conditioned at 53% relative humidity (RH), were placed in Payne permeability cups filled with 5 mL of distilled water (100% RH). Then, the cups were placed into desiccators containing Mg (NO_3_)_2_ over-saturated solution (53% RH), with a fan on top of each cup to decrease the resistance to water vapor transport, and weighed every 1.5 h in an analytical balance (±0.0001 mg). The WVP was calculated from the slope of the weight loss–time curves (water vapor transmission rate) considering the film thickness and the water vapor pressure gradient.

### 2.6. Oxygen Permeability

The oxygen permeability (*OP*) of the films, conditioned at 53% RH, was determined in duplicate, at 25 °C and 53% RH, using oxygen analyzer equipment (Systech, Model 8101e, Illinois IL, USA) according to ASTM F1927-14 [21]. The film samples were exposed to the oxygen flow, and the oxygen transmission rate was obtained every 15 min until equilibrium was reached. The *OP* values were calculated, taking the oxygen transmission rate and film thickness into account.

### 2.7. Thermal Properties

The thermal stability of the films was analyzed in duplicate using a thermogravimetric analyzer (TGA 1 Stare System analyzer, Mettler-Toledo, Greifensee Switzerland). Film samples (3–5 mg) were heated from 25 to 600 °C at 10 °C·min^−1^ under N_2_ flow at 10 mL·min^−1^. For each thermal event, the initial (*Tonset*) and maximum degradation rate (*Tpeak*) temperatures were determined by analyzing the thermogravimetric curves and their first derivate using STARe Evaluation Software (STARe SW 12.00, Mettler-Toledo, Greifensee Switzerland). 

The phase transitions of the polymer were analyzed, in duplicate, with a differential scanning calorimeter (DSC 1 Stare System analyzer, Mettler-Toledo, Greifensee Switzerland), operating under a nitrogen flow (10 mL·min^−1^). Samples of 5–10 mg were weighted in aluminum pans and heated from −40 to 200 °C at 10 °C·min^−1^; 1 min at 200 °C and then cooled to −40 °C at 10 °C·min^−1^; 2 min at −40 °C and then heated to 200 °C at 10 °C·min^−1^ (second heating step).

### 2.8. Color and Transmittance 

Optical properties of the films were obtained in triplicate, according to the Kubelka TGA 1 Stare System analyzer Munk theory of multiple scattering, to determine the film reflection spectra (from 400 to 700 nm) onto a black background (*R*_0_) and onto a white background (*R*) with reflectance (*R_g_*). The internal transmittance (*T_i_*) (Equation (1)) of the films was obtained to quantify the film transparency, and the infinite reflectance (*R_∞_*) (Equation (2)) was obtained to determine color coordinates. The film color coordinates L* (lightness), a* (redness–greenness), and b* (yellowness–blueness) were determined from *R_∞_*, considering the observer 10° and illuminant D65. The psychrometric coordinates hab* (Equation (5)) and Cab* (Equation (6)) and color difference (Equation (7)) with respect to the phenol-free film were also determined.

(1) $$T_{i}=\sqrt{(a+R_{o}^{2})-b^{2}}$$

(2) $$R_{\infty }=a-b$$

(3) $$a=\frac{1}{2}\left[R+\left(\frac{R_{o}-R+R_{g}}{R_{o}\cdot R_{g}}\right)\right]$$

(4) $$b=\sqrt{a^{2}-1}$$

(5) $$h_{ab}*=arctg(\frac{b*}{a*})$$

(6) $$C_{ab}*=\sqrt{a{*}^{2}+b{*}^{2}}$$

(7) $$\Delta E*=\sqrt{{\left(\Delta L*\right)}^{2}+{\left(\Delta a*\right)}^{2}+{\left(\Delta b*\right)}^{2}}$$

wherein $\Delta L*=L*-L_{o}*$; $\Delta a*=a*-a_{o}*$; $\Delta b*=b*-b_{o}*$; and *L*_0_*, *a*_0_*, and *b*_0_* are the color coordinates of the control film without phenolic compounds.

### 2.9. XDR Spectra

The X-ray diffraction spectra of the samples were obtained with an X-ray diffractometer (AXS/D8 Advance, Bruker, Karlsruhe, Germany) using Cu-Kα radiation (λ: 1.542 Å, 40 kV, 40 mA) at 40 kV and 40 mA, with a step size of 2.0°min^−1^ and a 2θ scanning angle between 5° and 50°. The data were obtained using XRD Commander (Bruker AXS GmbH, Karlsruhe, Germany) and processed with DIFFRAC.EVA (Bruker AXS GmbH, Karlsruhe, Germany) and DRXWin (Windows, version 2.3). The crystallinity index (CI) was calculated from the curves (Equation (8)) using the Origin 2020 software (OriginLab Corporation, Northampton, MA, USA).

(8) $$CI(\%)=\frac{A_{c}}{A_{c}+A_{a}}$$

wherein *A_C_* is the area of the crystalline peaks and *A_a_* is the area of the amorphous background.

### 2.10. FTIR Spectra

Film samples were characterized by the Fourier transform infrared spectra using the Agilent, Cary 630 FTIR (Santa Clara, CA, USA) equipment with attenuated total reflectance (ATR) mode from 4000 to 500 cm^−1^, with a resolution of 6 cm^−1^ and 128 scans. The data were processed by the Origin 2020 software (OriginLab Corporation, MA, USA).

### 2.11. Stability of Phenolic Compounds in the Film

The total content of phenolic compounds in the films was quantified in newly formed films and those stored for 102 days at 0% RH and 25 °C. This was carried out by methanol extraction and subsequent spectrophotometric determination [22]. For this purpose, film samples (100 mg) were cut into thin strips and immersed in 10 mL of methanol under stirring for 48 h at 20 °C. Absorbance measurements of the methanolic extracts were obtained at 321, 308, and 291 nm, respectively, for ferulic acid, vanillin, and catechin, using a UV–visible spectrophotometer (Thermo Scientific Evolution 201, Waltham, MA, USA). Calibration curves were previously obtained for the methanolic solutions of each pure phenolic compound. In all cases, the methanolic extract of the active-free PHBV films was used as a blank. The measurements were taken in quadruplicate for each film sample.

### 2.12. Overall Migration

The overall migration (*OM*) of the films was determined in triplicate according to the UNE-EN 1186-3:2023 [23] standards. For this, 1 dm^2^ of film samples was immersed in 100 mL of simulants and kept at 40 °C for 10 days. Afterward, the films were removed, the simulant evaporated until dryness, and the *OM* was determined from the migrated dried mass, as described in the standard. The simulants used were simulant A (distilled water), simulant B (3% (*w*/*v*) acetic acid), and simulant C (10% (*v*/*v*) ethanol) to simulate aqueous foods, and ethanol 95% (*v*/*v*) to simulate fatty foods. 

The specific migration of the incorporated phenolic acids was also measured by extracting the migrated dried residue with 10 mL of methanol for 48 h under stirring and then measuring the absorbance in a spectrophotometer (Thermo Scientific Evolution 201, Waltham, MA, USA) at the absorption maximum wavelength of each phenolic compound (321 nm for ferulic acid, 308 nm for vanillin, and 291 nm for catechin). The concentration of phenolic compounds was determined with the respective calibration curves and referred to per mass unit of the film. The amount of non-active compounds migrated in each case was determined by subtracting the migrated amount of phenolic from the corresponding total migrated mass of each film. 

## 3. Results and Discussion

### 3.1. Film Microstructure

**Figure 1:**
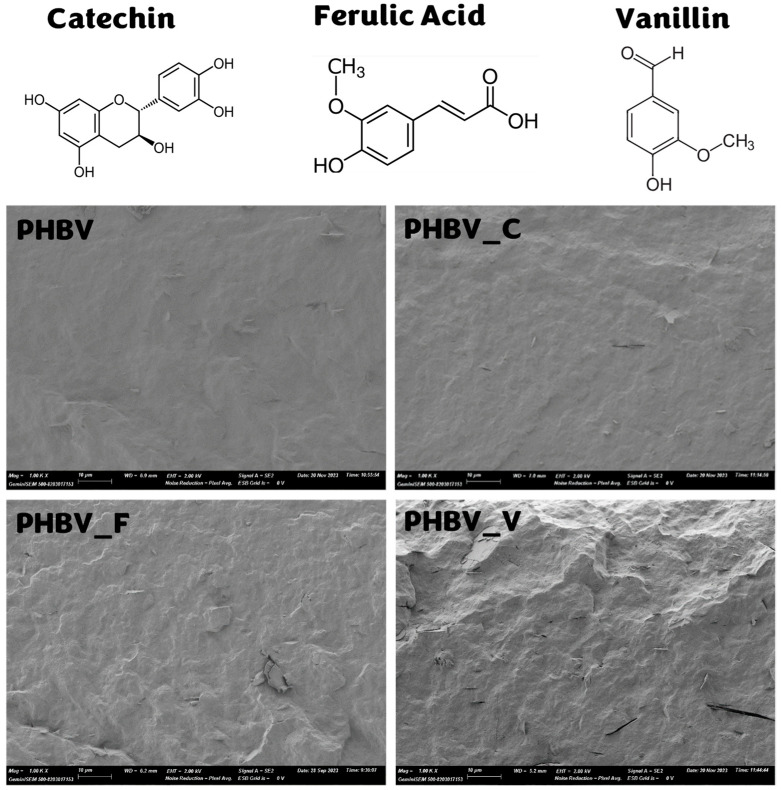
FESEM micrographs of the cross-section of PHBV films with the different phenolic compounds (molecular structure shown).

Figure 1 shows the FESEM micrographs of the cross-section (1000×) of the different film samples where their morphology can be observed. A homogenous structure can be appreciated for every sample with no dispersed particles, thus indicating the good integration of phenolic compounds within the PHBV matrix during melt blending. Similar homogeneous structures for neat PHBV films were reported in previous studies [3,24], with smooth and uniform cross-section micrographs containing some micro-flakes attributed to the presence of nucleating agents in commercial PHBV [25]. The good integration of phenolic compounds within the PHBV matrix can be attributed to their relatively low melting temperature (175, 173, and 84 °C) for catechin, ferulic acid, and vanillin, respectively, which allows for their melting and homogenous mixing during the melt blending with the polymer at 170 °C. Likewise, as reported by other authors [3,24], phenolic compounds can form hydrogen bonds with carbonyl groups of polyesters, which allows for their bonding to the polymer chains, thus contributing to the homogenous blending.

### 3.2. Tensile and Barrier Properties of the Films

As shown in Table 1, no difference in the thickness of the different films was observed, which indicates a similar flowability of the materials during the thermoprocessing. Tensile properties of net PHBV films were in the range reported by other authors, with high stiffness and low stretchability. In terms of stiffness and strength, they are comparable to polypropylene (PP) [25]. In general, the incorporation of phenolic compounds provoked a decrease in the values of the tensile strength (*TS*), elongation at break (ε%), and elastic modulus (*EM*) due to the reduction in the interchain forces, weakening the cohesion strength of the matrix. Nevertheless, catechin enhanced the *EM* of the films, which could be attributed to a cross-linking effect favored by the hydroxyl positions in the molecular structure (Figure 1), allowing for its hydrogen bonding to the carbonyl groups of different polymer chains. This promoted the interchain crosslink with the subsequent improvement of the matrix toughness, as also observed by other authors for tea polyphenols in PHBV matrices [26]. The cross-linking effect of ferulic acid has also been reported in polyester films, but overlapped with a certain hydrolytic effect of the polymer chains that reduced the matrix cohesion forces [22]. Consequently, films with ferulic acid had lower *EM* values but similar ε% and *TS* values to those containing catechin. Similar behavior of ferulic acid into the PHBV matrix was already described by other authors [3,27]. Meanwhile, vanillin reduced the fracture resistance and elastic modulus, without notable changes in the film extensibility, compared to pure PHBV films, which can be mainly attributed to a plasticizing effect of the phenolic compound, as previously reported for PHBV films with vanillin [12].

Concerning the water vapor permeability (*WVP*) of the films, PHBV film exhibited values in the range reported by other authors [28,29,30], being similar to those of polyethylene terephthalate (PET) [25]. *WVP* values significantly increased in the films with vanillin (PHBV_V), which can be attributed to its plasticizing effect that increases the molecular mobility, promoting the diffusion of water molecules into the matrix and thus the permeation rate. However, no statistically significant differences were observed for PHBV films and those containing catechin (PHBV_C) and ferulic acid (PHBV_F), which could be explained by the anti-plasticizing effect caused by the cross-linking of molecular chains that offsets the expected reduction in the interchain forces produced by an incorporated compound in the polymer matrix. This cross-linking effect contributes to reducing the molecular mobility in the matrix, limiting diffusional processes. For the PHBV_F film, this effect was more attenuated, probably due to the hydrolytic capacity of the acid that reduces the polymer chain length and increases the permeation capacity of the matrix, in comparison to the PHBV_C film.

The oxygen permeability (*OP*) of PHBV films was also in the range reported by other authors [3,28,29]. In general, the *OP* values decreased with the incorporation of phenolic compounds into the polymer matrix, which can be attributed to the oxygen-scavenging effect of these compounds [22,31] coupled with the plasticizing or anti-plasticizing effect of each phenolic compound, which modifies the molecular diffusion of the gas. Indeed, the lowest *OP* values were obtained for the catechin-containing films (PHBV_C), whereas the highest values were for films with vanillin (PHBV_V), exhibiting a cross-linking and plasticizing effect, respectively. 

### 3.3. Thermal Behavior of the Films 

Table 2 shows the thermal parameters obtained from DSC curves (first and second heating scan) of the PHBV films with or without phenolic compounds. In the first scan, the thermoprocessing history of the material is reflected, while the second scan reveals the behavior of the material after cooling at 10 °C/min, which promotes the crystallization of the polymer under more controlled conditions. Thus, some differences were observed in the parameters from the first and second scans, where the polymer glass transition and melting endotherm were observed. 

Glass transition temperature (*T_g_*) was higher for films with ferulic acid (PHBV_F) and catechin (PHBV_C) than for phenol-free PHBV. In contrast, the incorporation of vanillin (PHBV_V) decreased the *T_g_* with respect to the PHBV film. This agrees with the different interactions of the phenolic compounds with the polymer chains, depending on their molecular structure, as commented on above. Ferulic acid and catechin provoked an anti-plasticizing effect associated with the cross-linking of the polymer chains with phenolic compounds that reduces the molecular mobility in the matrix, thus increasing the *T_g_*. The cross-linking effect of phenol structures in PHBV matrices has been previously reported, highly depending on the molecular structure and stereochemistry [32]. Vanillin molecules only possess one hydroxyl group, so their ability to interchain bond is limited. Thus, PHBV_V films were more plasticized than PHBV films, exhibiting lower *T_g_* values due to their interrupting effect on the chain associations, weakening the cohesion forces of the polymer matrix. 

The melting temperature (*T_m_*) of PHBV was significantly decreased by the incorporation of phenolics, both in the first and second heating scans, as previously observed by other authors for phenolic acids [3,31]. This suggests the presence of smaller crystalline formations that melted at lower temperatures. Likewise, a tendency to decrease in the melting enthalpy and crystallization degree was also observed with the incorporation of the phenolic compounds. These effects could be attributed to the blending effect and interactions of phenolics with the polymer chains through the hydrogen bond formation between phenolic hydroxyls and polyester carbonyls. This makes the polymer crystallization more difficult, resulting in a slightly reduced size of crystals and more limited crystallization. Nevertheless, the crystallinity reduction was not statistically significant and so it will not have a notable effect on the material performance. The crystallinity percentage was estimated from the melting enthalpy, considering a **∆***H_m_* value of 132 J/g polymer for completely crystalline PHBV [3].

**Figure 2:**
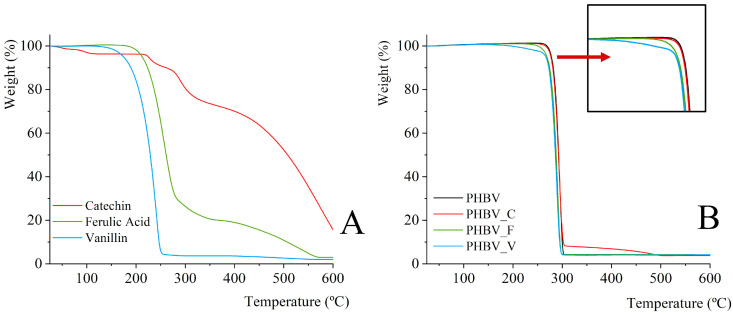
TGA curves of (**A**) pure phenolic compounds and (**B**) the different PHBV films containing or not containing 5% wt. of phenolics (catechin: C, ferulic acid: F, or vanillin: V).

Concerning the thermal stability of the materials, Figure 2 shows the TGA curves of the pure phenolic compounds and the respective films of PHBV containing these phenols. Pure phenolics exhibited similar TGA curves to those described for catechin [33], vanillin [34], and ferulic acid [35,36], respectively. Vanillin was the most thermo-sensitive compound, starting its degradation near 150 °C and reaching the maximum degradation rate at 220 °C. Ferulic acid started degradation near 250 °C, while catechin showed greater thermal stability, exhibiting a first weight loss step near 100 °C, corresponding to the loss of bonded water (close to 4%) and successive degradation steps from about 250 °C. These results suggest that vanillin could become more altered during the film thermoprocessing than ferulic acid and catechin. The incorporation of phenolic compounds hardly impacts the thermal stability of the polymer, which showed the typical thermo-degradation curve shown in Figure 2B, previously described in other studies [37,38], with onset temperature at 275 °C and the maximum degradation rate at 293 °C. Nevertheless, ferulic acid, and mainly vanillin, slightly decreased the onset temperature of degradation of PHBV while the PHBV_V sample exhibited a small weight loss after about 150 °C that can be attributed to the partial thermal degradation of the incorporated vanillin. The decrease in the onset temperature provoked by vanillin could be attributed to its plasticizing effect that weakens the interchain forces of the polymer, making it less thermostable. Xavier et al. [12] also observed an initial weight loss step at 150 °C in PHB films with vanillin, attributed to the thermal sensitivity of vanillin. Ferulic acid could provoke the partial hydrolysis of the PHBV chains, giving rise to oligomers of lower molecular weight with a lower degradation temperature. In contrast, the most thermostable catechin does not cause notable changes in the thermal stability of the polymer, giving rise to a greater mass residue at *T* > 300 °C, attributed to the final degradation of this incorporated phenolic compound. 

### 3.4. Color and Transmittance of the Films

**Figure 3:**
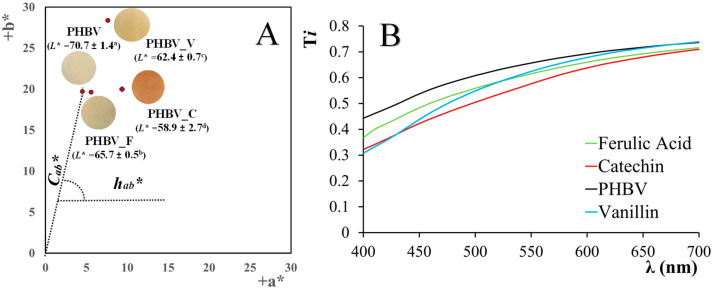
(**A**) Color coordinates (lightness: L*, chrome: C_ab_*, and hue: h_ab_*) and (**B**) internal transmittance of PHBV films containing or not containing 5% wt. of phenolics (catechin: C, ferulic acid: F, or vanillin: V). Note. In Figure A, different superscript letters indicate significant differences between different samples (Tukey test, *p* < 0.05).

Figure 3 shows the images of the PHBV films with and without the different phenolic compounds, reflecting the slight color changes caused by the compound incorporation (Figure 3A) and the small reduction in the film transparency (Figure 3B). These changes were quantified through the color coordinates (L*, C_ab_*, and h_ab_*) shown in Figure 3A and total color difference (ΔE*) induced by phenolic compounds with respect to the phenol-free film. 

The neat PHBV films exhibited the greatest lightness, with a more yellowish and less saturated color. Ferulic acid had the lowest impact on the film color, slightly reducing the lightness and hue value, as previously reported by other authors [3]. In contrast, the films with vanillin (PHBV_V) exhibited the highest color saturation while catechin-containing films were the darkest and reddest samples. Catechin and vanillin provoked the highest color difference with respect to the PHBV films (∆E* = 13.2 and 12.4, respectively), while ferulic acid led to the lowest color difference (∆E* = 5.1). Similar effects were observed for catechin when incorporated into PVA–starch films [15]. The induced changes in the film color by phenolic compounds are related to their own natural color and potential interactions with the polymer matrix.

### 3.5. X-ray Diffractograms and FTIR Spectra of the Films

**Figure 4:**
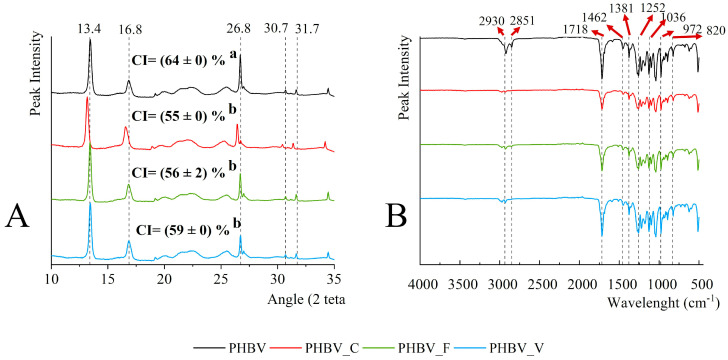
X-ray diffractograms (**A**) and FTIR spectra (**B**) of PHBV films containing or not containing 5% wt. of phenolics (catechin: C, ferulic acid: F, or vanillin: V). Note. Different superscript letters indicate significant differences between different samples (Tukey test, *p* < 0.05).

Figure 4A shows the X-ray diffractograms of PHBV films. All samples presented the characteristic crystallization pattern of PHBV in an orthorhombic lattice [39] with typical diffraction peaks at 2θ: 13.4, 16.8, 26.8, and 30 [40,41] assigned to the (020), (110), (101), and (002) diffraction planes. Ponjavic et al. [42] reported an additional peak at 31.7, corresponding to the (200) diffraction plane that also appeared in the obtained diffractograms. 

The crystallinity index (*CI*) obtained by Equation (8) was also shown in Figure 4A and was significantly higher for net PHBV films than for those containing phenolic compounds. This agrees with the crystallinity degree deduced from DSC analysis, with the expected differences due to the use of dynamic (DSC) or static (X-ray spectra) techniques. Therefore, the incorporation of phenolic compounds affected the PHBV crystallization kinetics, hindering the crystal growth, thus reducing the size of crystalline domains and final crystallinity, as reported by other authors for different phenolic compounds [1,3,26].

Figure 4B shows the FTIR spectra of the PHBV films that exhibited the typical absorption reported for PHBV [3,24]. The bands appearing at 2930 cm^−1^ and 2851 cm^−1^ correspond to the C–H symmetric stretching vibration of CH_3_ of PHBV [39,42]. The intense PHBV absorption peak associated with the carbonyl group (C=O) stretching vibrations appears at 1720 cm^-1^ [39,42]. The bands around 1462 cm^-1^ and 1382 cm^−1^ are associated with the bending vibration modes of the C–H, while bands at 1259 cm^−1^ and 1036 cm^−1^ correspond to the stretching vibration of the C–O–C bond [39,42]. Finally, bands that appear at 972 to 820 cm^−1^ are attributed to the stretching vibrations of the C–H bonds [3]. The typical vibration bands of phenolic compounds hardly appeared in the spectra of phenol-containing films due to the low content of these compounds. Likewise, no significant shift in the vibration band of carbonyl at 1720 cm^−1^ was observed due to the hydrogen bond formation with the phenolic hydroxyls. As reported by other authors [24], this shift is slight and difficult to observe in the FTIR spectrum.

### 3.6. Retention of Active Compounds in the Films and Overall Migration

**Figure 5:**
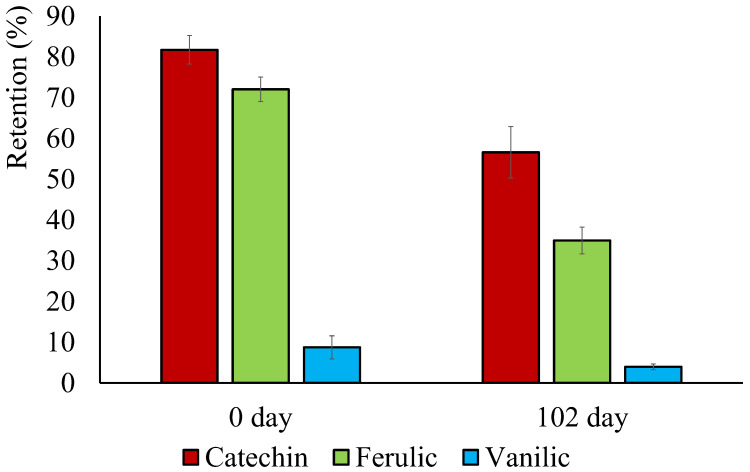
Retention percentage (final amount with respect to that initially incorporated) of phenolic compounds within the PHBV films for newly prepared samples and after storage at 0% relative humidity and 25 °C for 102 days.

The incorporated phenolic compounds could be partially degraded during the film thermoprocessing or altered throughout time depending on the storage conditions. The losses of active compounds during the film processing were evaluated through their quantification in the film by methanol extraction and spectrophotometric determination. Figure 5 shows the retention percentage (final extracted amount with respect to that initially incorporated) of each phenolic compound in the newly prepared films. These values were 82%, 72%, and 7% for catechin, ferulic acid, and vanillin, respectively. Thus, the compound losses were coherent with the different thermo-sensitivities of the compounds observed in the TGA analyses and the film processing temperature. Vanillin was the compound with the greatest losses according to its lower thermo-degradation temperature. Similar losses of ferulic acid were previously reported during melt blending with PHBV at similar temperatures [9]. Catechin was the least degraded compound, consistent with its higher degradation temperature (Figure 2A), resulting in higher retention values. The obtained retention values could be affected by the uncomplete compound extraction with methanol at 20 °C (Section 2.11) since the extraction method was not optimized and a part of the compound could still be present in the methanol-extracted film. In fact, several studies pointed out that the temperature increase highly promoted the extraction yield of vanillin [43].

After 102 storage days, greater losses of active compounds were observed in every case, which suggests that the compounds are progressively oxidized in contact with the atmosphere conditions. Phenolic compounds are prone to oxidization due to their reducing nature, depending on the environmental conditions, such as light exposure, pH, oxygen pressure, temperature, and time [44]. In fact, some authors reported that catechin degrades over time, even at room temperature [45], while ferulic acid and vanillin suffer photo-oxidation at room temperature [46]. All of this could result in the reduction of compound concentration in the films over time.

Relevant information for developed packaging materials intended for food contact is the migration of the material compounds into the food. Article 12 of the European Regulation (EC) 10/2011 specifies both the overall migration limit (*OML* = 10 mg/dm^2^) and the assay conditions (food simulants, temperature, and contact time) to quantify the overall migration (*OM*). For packaging materials with active compounds, Regulation (EC) No 450/2009 establishes that the amount of active components migrated should not be considered for determining the *OM* values since they are not considered as a part of the potentially dangerous passive material, while their migration is required to exert their active role. Therefore, the fraction of the migrated compounds corresponding to the phenolic compounds (catechin, ferulic acid, or vanillin) was determined (specific migration) and subtracted from the total migrated residue for each simulant. Nevertheless, as established in Regulation (EC) No 1935/2004, the use of active compounds for food contact materials should be previously authorized by the European Food Safety Authority (EFSA).

**Figure 6:**
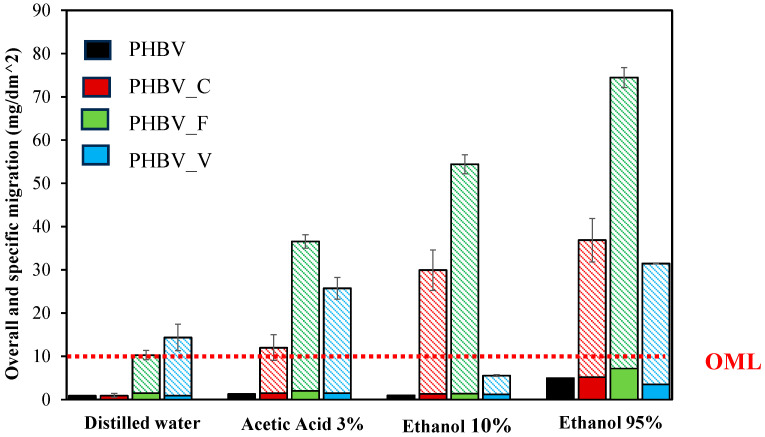
Migration (mg/dm^2^) of passive material (solid bars) and active phenols (non-solid bars) in different food simulants from PHBV films containing or not containing 5% wt. of phenolics (catechin: C, ferulic acid: F, or vanillin: V). The overall migration limit (*OML*) for passive material is marked (dashed line).

Figure 6 shows the *OM* values of passive material (solid bars) and the specific migration of phenolics (non-solid bars) from the PHBV films in different food simulants. For neat PHBV, *OM* values were lower than the *OM* limit (OML: 10 mg/dm^2^) in every food simulant, as reported in other studies [2,47]. For films containing phenolic compounds, *OM* values of passive material were also lower than the OML but, in general, these were slightly higher than for net PHBV films. This mainly occurred for films containing ferulic acid and could be attributed to the hydrolytic effect of ferulic acid that produces oligomers with a higher migration capacity. The greatest *OM* values were obtained in the ethanol-rich simulant due to the swelling and degradation ability of ethanol, which modifies the PHBV matrix, affecting the compound release [3,48]. 

**Figure 7:**
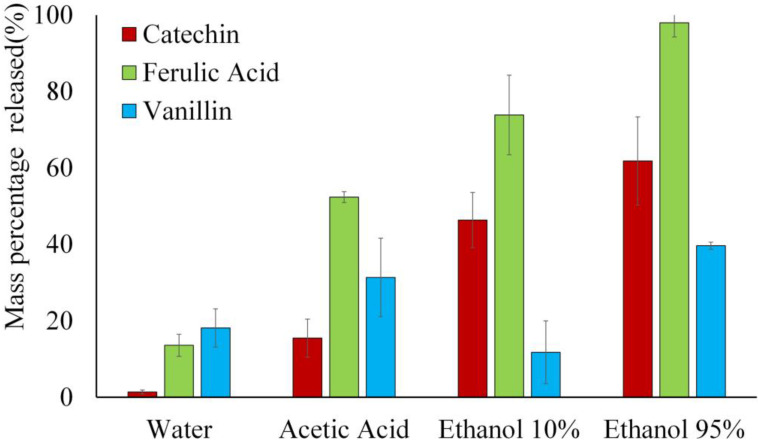
Mass percentage of the different phenolic compounds released into the different food simulants with respect to those initially incorporated into the PHBV films (5% wt.).

Concerning the specific migration of phenolic compounds (non-solid bars in Figure 6), catechin exhibits relatively low values in every simulant despite its higher retention in the film, which agrees with the above-described cross-linking effect that limits its diffusion through the film and migration into the simulants. The amount migrated increased as the ethanol ratio rose in the simulant due to the polymer swelling and degradation, which favors the compound release [15]. Ferulic acid exhibited, in general, the highest migration values in every simulant, increasing in acid water and when the ethanol ratio rose, as described in previous studies [3]. Except in distilled water, vanillin exhibited the lowest migration levels, partly due to its higher degradation during the film processing and lower final concentration in the films. Its major migration also occurred in ethanol 95%, as observed in other studies with PHB films [12]. Figure 7 shows the mass percentage of migrated phenolics in each simulant with respect to the initial amount incorporated into the films. In ethanol 95%, practical total migration of the incorporated ferulic was observed, while only 40 and 62% of the incorporated amount of vanillin and catechin, respectively, migrated. These values indicate that more vanillin than that quantified by methanol extraction was present in the films, whereas the catechin migrated amount (62%) was lower than that present in the film (about 85%).

Therefore, the incorporation of phenolic compounds slightly affected the migration of passive material of PHBV films, regardless of the specific migration of each compound that was greatly affected by the simulant polarity (ethanol concentration) or pH and by the available compound concentration in the film. In this sense, catechin, with a great bonding capacity with the polymer chains, migrated to a lower extent than ferulic acid or vanillin. The active properties of the material will be affected by the amount of phenol released to the food surface, which, in turn, will depend on the phenol concentration in the film, its bonding forces with the matrix, and the nature of the food in contact, all affecting the release ratio.

## 4. Conclusions

The incorporation of phenolic compounds with different molecular structures into the PHBV films to confer them antioxidant and/or antimicrobial activity caused modifications in the properties of the matrix due to the different molecular interactions with the polymer chains. Ferulic acid, and mainly catechin, produced a cross-linking effect in the polymer matrix, while vanillin had a plasticizing effect, as deduced from the glass transition temperature of the amorphous phase and the effect on the tensile and barrier properties of the material. All compounds increased the oxygen barrier capacity of PHBV films due to their antioxidant capacity, which provides the films with oxygen scavenging capacity. Likewise, all phenolic compounds decreased the melting temperature and enthalpy of PHBV, interfering with the polymer crystallization. These compounds slightly modified the color and transparency of the films, making them slightly darker, with greater color saturation and a redder hue, especially for catechin and vanillin, due to the natural coloration of the compounds. The overall migration test revealed that the active films could be used as food contact materials since the OML was not exceeded in every food simulant, while the phenolic compounds were released depending on the content in the film, bonding forces with the polymer matrix, and simulant polarity. Therefore, the incorporation of phenolic compounds into the PHBV matrix did not have a negative effect on its properties, conferring to the films an additional function as antioxidant/antimicrobial materials. Nevertheless, the thermo-sensitivity of the phenolic compound and its ability to interact with the polymer chains must be considered to ensure its preservation during the material thermo-processing and its final release into the food substrate. In this sense, catechin or ferulic acid would be preferably recommended, but further studies on real foods must be carried out to validate these materials in food packaging applications.

## Figures and Tables

**Table 1:** Thickness, tensile strength (*TS*), elongation at break (*ε*), elastic modulus (*EM*), water vapor permeability (*WVP*), and oxygen permeability (*OP*) for films containing or not containing 5% wt. of different phenolics (catechin: C, ferulic acid: F, or vanillin: V).

Material	Thickness (mm)	*TS* (MPa)	*ε* (%)	*EM* (MPa)	*WVP* × 10^12^ (g/Pa·s·m)	*OP* × 10^12^ (cm^3^/Pa·s·m)
PHBV	0.130 ± 0.013 ^a^	32 ± 3 ^a^	2.0 ± 0.2 ^a^	2000 ± 90 ^b^	3.3 ± 0.4 ^b^	0.220 ± 0.040 ^a^
PHBV_C	0.129 ± 0.011 ^a^	17 ± 2 ^c^	1.0 ± 0.1 ^b^	2200 ± 60 ^a^	3.0 ± 0.2 ^b^	0.070 ± 0.003 ^c^
PHBV_F	0.124 ± 0.010 ^a^	14 ± 2 ^c^	1.2 ± 0.4 ^b^	1800 ± 90 ^c^	3.2 ± 0.4 ^b^	0.150 ± 0.030 ^b^
PHBV_V	0.124 ± 0.013 ^a^	23 ± 2 ^b^	1.8 ± 0.3 ^a^	1500 ± 110 ^d^	5.0 ± 1.0 ^a^	0.210 ± 0.012 ^ab^

Note. Different superscript letters indicate significant differences between different samples (Tukey test, *p* < 0.05).

**Table 2:** Glass transition temperature (*T_g_*), melting temperature (*T_m_*), enthalpy (**∆***H_m_*), and crystallinity (*XC*) obtained from DSC thermograms of the 1st and 2nd heating scans for the different PHBV films containing or not containing 5% wt. of different phenolics (catechin: C, ferulic acid: F, or vanillin: V).

Material	First Heating				Second Heating			
	*T_g_* (°C)	*T_m_* (°C)	∆*H_m_* (J/g PHBV)	*XC* (%)	*T_g_* (°C)	*T_m_* (°C)	∆*H_m_* (J/g PHBV)	*XC* (%)
PHBV	6.0 ± 0.0 ^c^	170.1 ± 1.0 ^a^	85.0 ± 4.3 ^a^	64.4 ± 3.3 ^a^	6.0 ± 1.0 ^c^	170.4 ± 1.0 ^a^	92.4 ± 4.4 ^a^	70.0 ± 3.3 ^a^
PHBV_C	29.2 ± 0.2 ^a^	168.0 ± 0.3 ^b^	84.0 ± 1.2 ^a^	63.5 ± 0.9 ^a^	24.0 ± 1.0 ^a^	166.0 ± 0.3 ^b^	86.3 ± 1.2 ^a^	65.4 ± 1.0 ^a^
PHBV_F	11.2 ± 0.3 ^b^	167.0 ± 0.4 ^c^	82.0 ± 6.3 ^a^	61.7 ± 4.7 ^a^	10.0 ± 1.0 ^b^	166.0 ± 0.3 ^b^	89.2 ± 6.0 ^a^	68.0 ± 4.2 ^a^
PHBV_V	3.4 ± 0.1 ^d^	165.2 ± 1.0 ^d^	82.1 ± 2.0 ^a^	62.2 ± 1.1 ^a^	5.0 ± 1.2 ^c^	165.0 ± 1.0 ^b^	90.4 ± 2.0 ^a^	69.0 ± 1.3 ^a^

Note. Different superscript letters indicate significant differences between different samples (Tukey test, *p* < 0.05).

## Data Availability

The raw data supporting the conclusions of this article will be made available by the authors on request.
